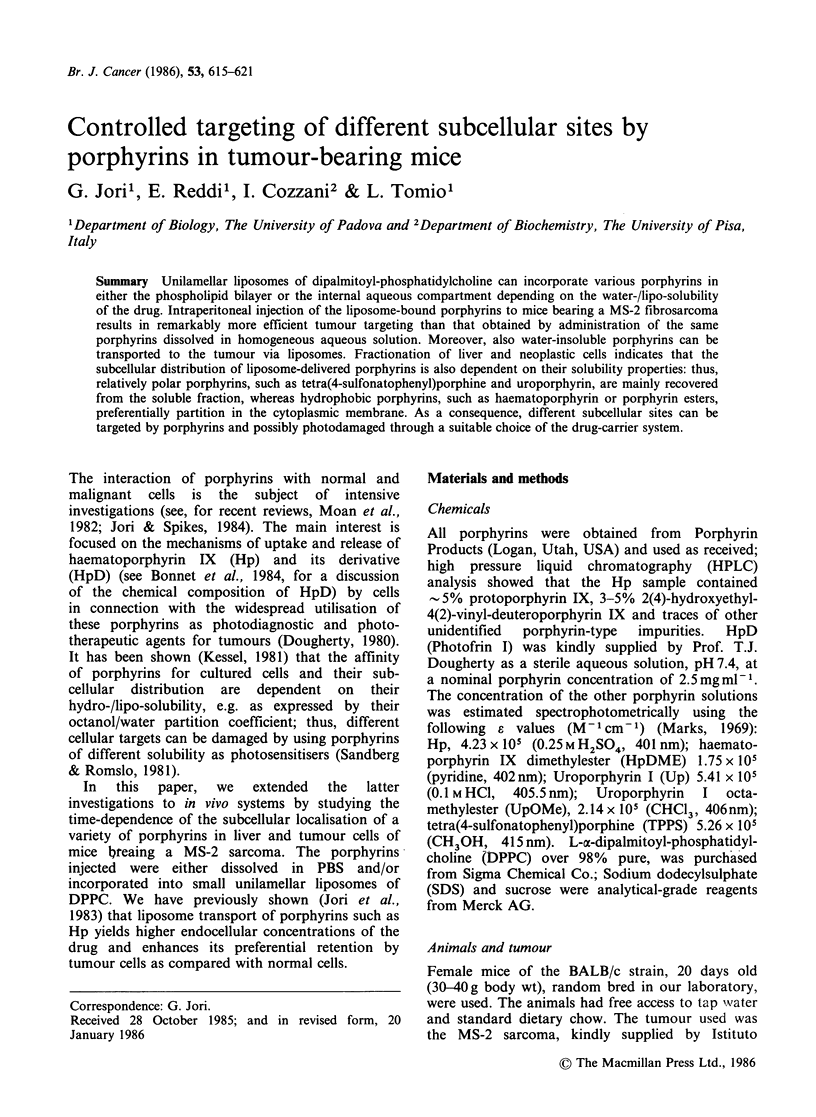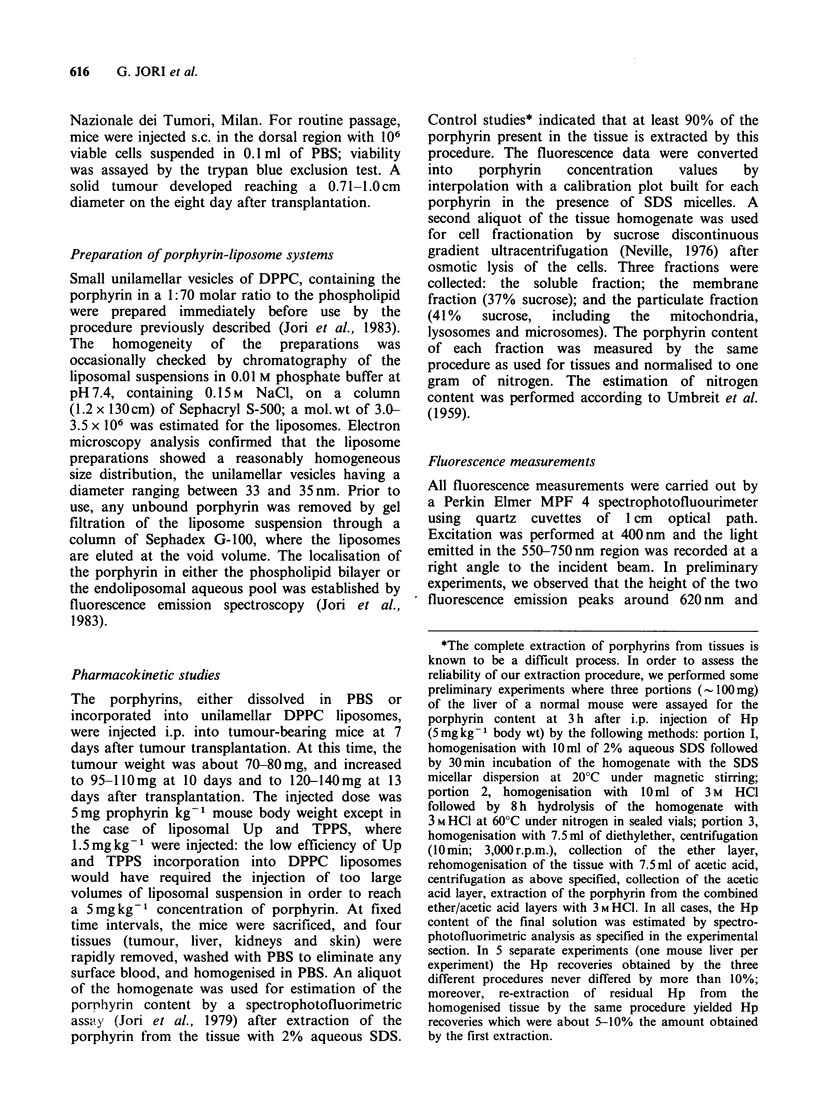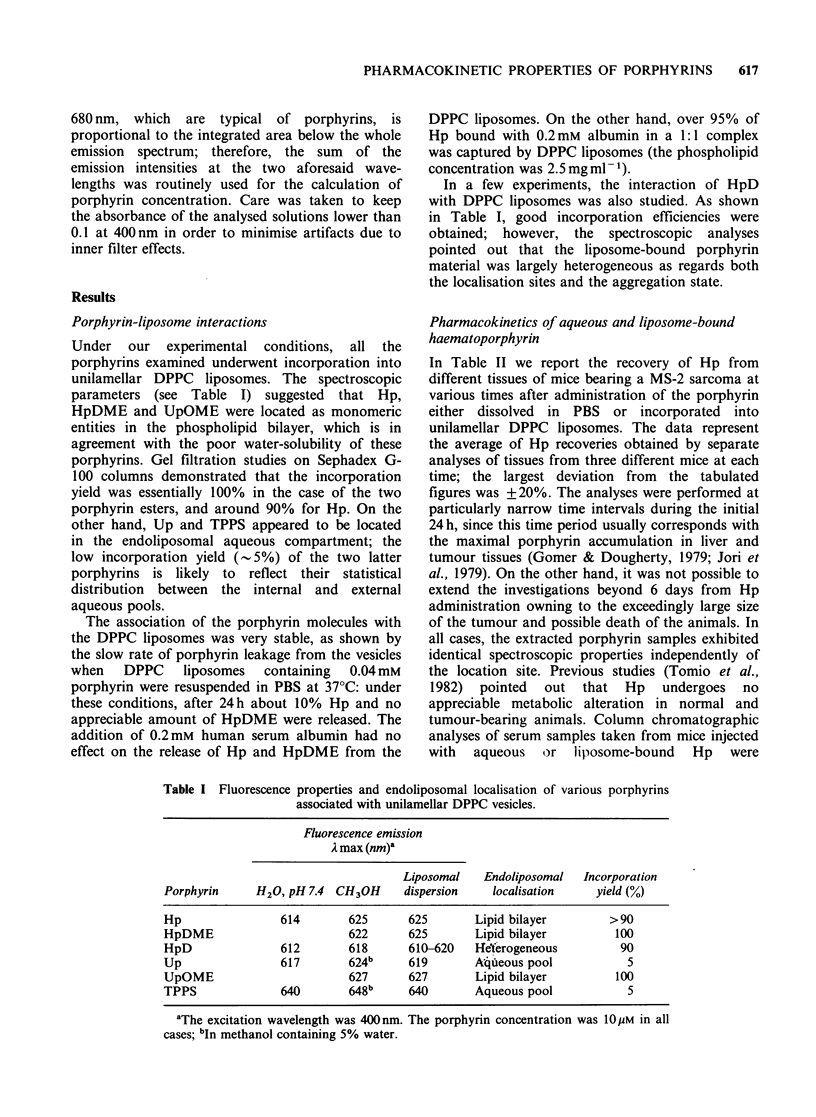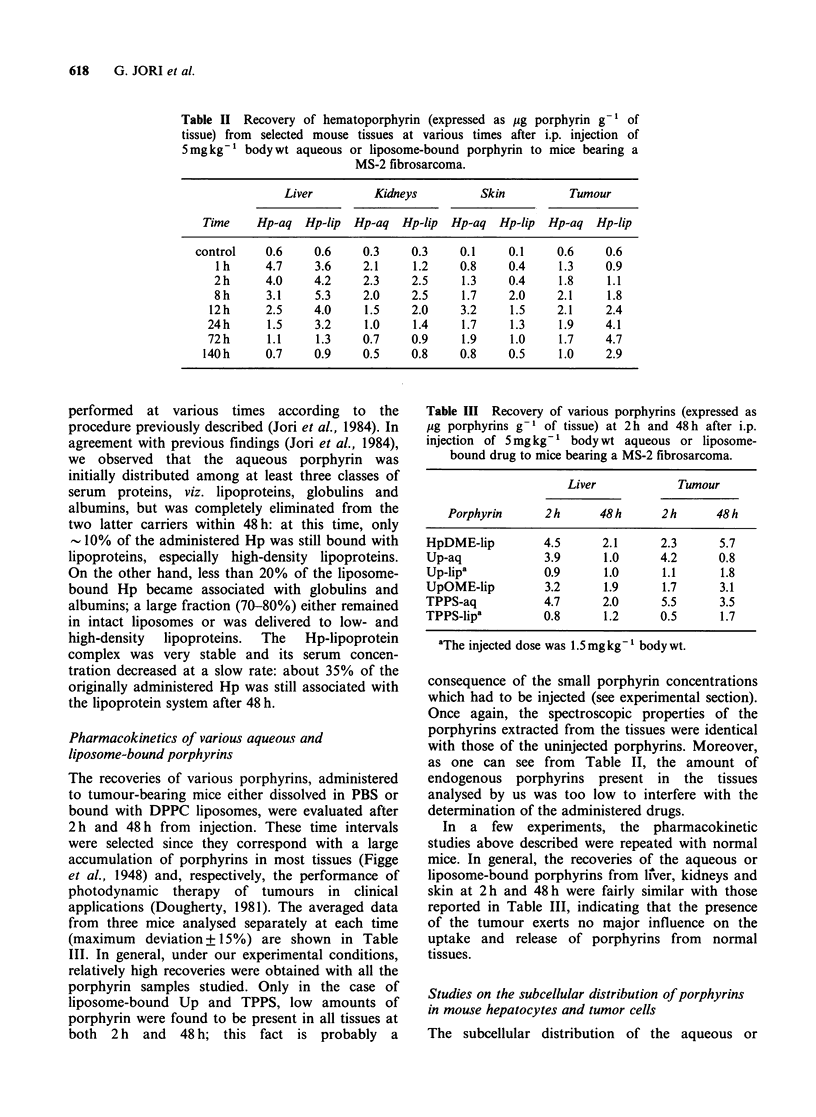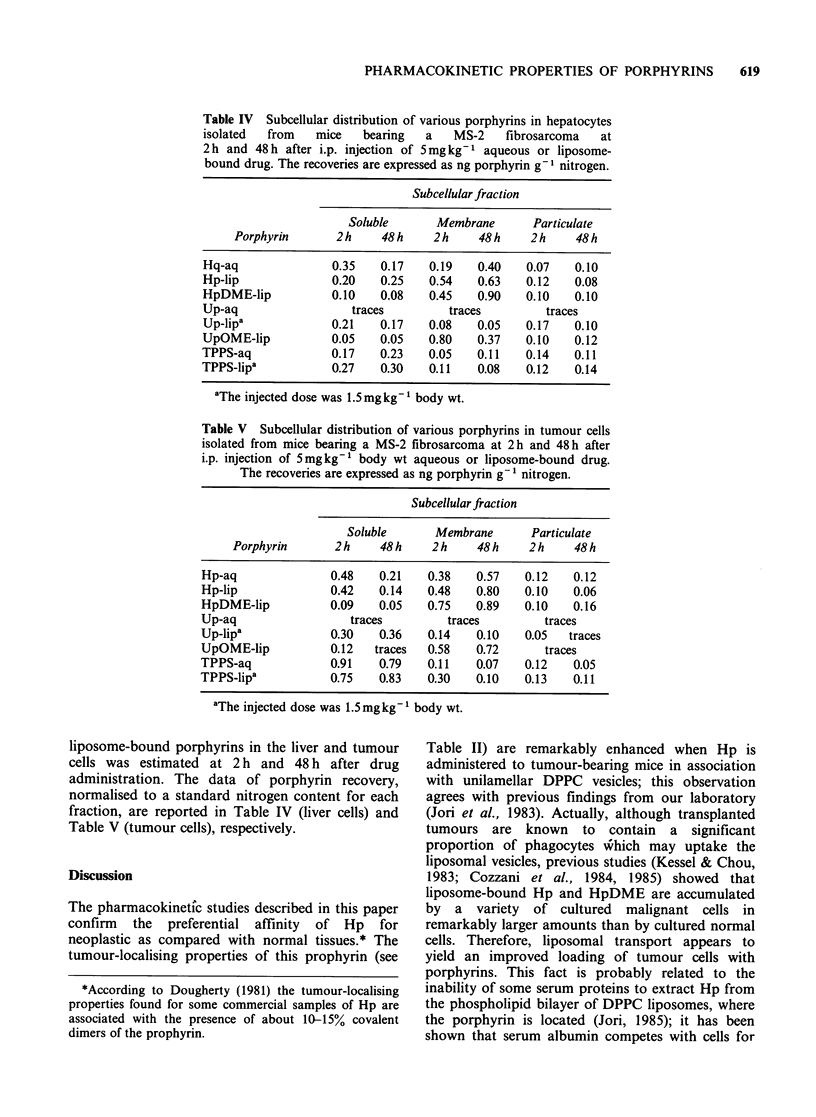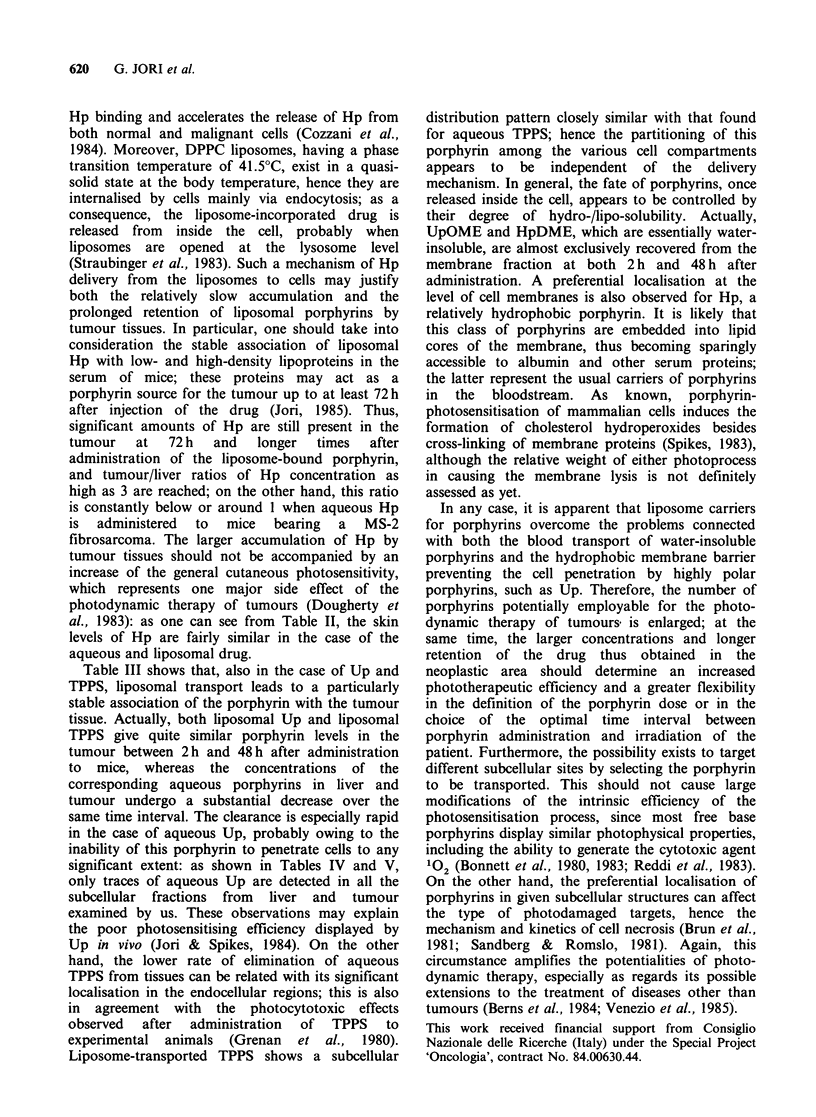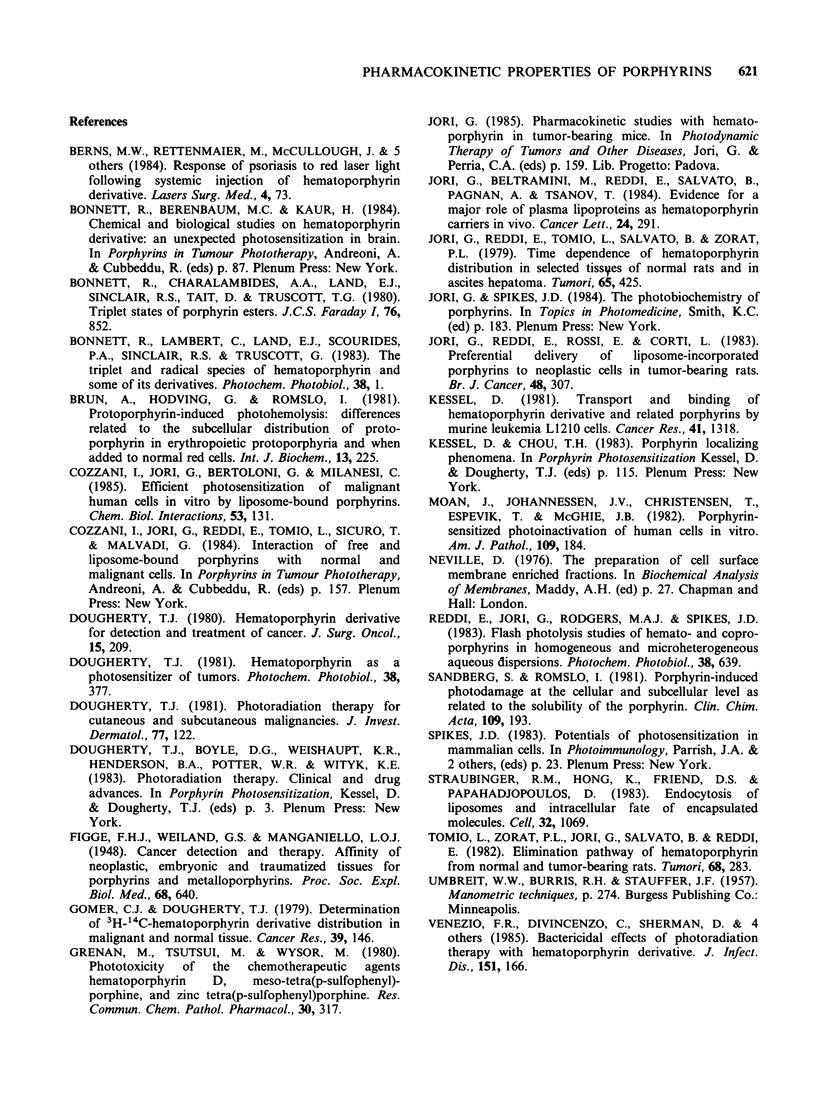# Controlled targeting of different subcellular sites by porphyrins in tumour-bearing mice.

**DOI:** 10.1038/bjc.1986.104

**Published:** 1986-05

**Authors:** G. Jori, E. Reddi, I. Cozzani, L. Tomio

## Abstract

Unilamellar liposomes of dipalmitoyl-phosphatidylcholine can incorporate various porphyrins in either the phospholipid bilayer or the internal aqueous compartment depending on the water-/lipo-solubility of the drug. Intraperitoneal injection of the liposome-bound porphyrins to mice bearing a MS-2 fibrosarcoma results in remarkably more efficient tumour targeting than that obtained by administration of the same porphyrins dissolved in homogeneous aqueous solution. Moreover, also water-insoluble porphyrins can be transported to the tumour via liposomes. Fractionation of liver and neoplastic cells indicates that the subcellular distribution of liposome-delivered porphyrins is also dependent on their solubility properties: thus, relatively polar porphyrins, such as tetra(4-sulfonatophenyl)porphine and uroporphyrin, are mainly recovered from the soluble fraction, whereas hydrophobic porphyrins, such as haematoporphyrin or porphyrin esters, preferentially partition in the cytoplasmic membrane. As a consequence, different subcellular sites can be targeted by porphyrins and possibly photodamaged through a suitable choice of the drug-carrier system.


*Br. J. Cancer* (1986), **53**, 615–621

### Controlled targeting of different subcellular sites by porphyrins in tumour-bearing mice

G. Jori[1], E. Reddi[1], I. Cozzani[2] & L. Tomio[1]

[1]*Department of Biology, The University of Padova and* [2]*Department of Biochemistry, The University of Pisa, Italy*

**Summary** Unilamellar liposomes of dipalmitoyl-phosphatidylcholine can incorporate various porphyrins in either the phospholipid bilayer or the internal aqueous compartment depending on the water-/lipo-solubility of the drug. Intraperitoneal injection of the liposome-bound porphyrins to mice bearing a MS-2 fibrosarcoma results in remarkably more efficient tumour targeting than that obtained by administration of the same porphyrins dissolved in homogeneous aqueous solution. Moreover, also water-insoluble porphyrins can be transported to the tumour via liposomes. Fractionation of liver and neoplastic cells indicates that the subcellular distribution of liposome-delivered porphyrins is also dependent on their solubility properties: thus, relatively polar porphyrins, such as tetra(4-sulfonatophenyl)porphine and uroporphyrin, are mainly recovered from the soluble fraction, whereas hydrophobic porphyrins, such as haematoporphyrin or porphyrin esters, preferentially partition in the cytoplasmic membrane. As a consequence, different subcellular sites can be targeted by porphyrins and possibly photodamaged through a suitable choice of the drug-carrier system.

The interaction of porphyrins with normal and malignant cells is the subject of intensive investigations (see, for recent reviews, Moan *et al.*, 1982; Jori & Spikes, 1984). The main interest is focused on the mechanisms of uptake and release of haematoporphyrin IX (Hp) and its derivative (HpD) (see Bonnet *et al.*, 1984, for a discussion of the chemical composition of HpD) by cells in connection with the widespread utilisation of these porphyrins as photodiagnostic and phototherapeutic agents for tumours (Dougherty, 1980). It has been shown (Kessel, 1981) that the affinity of porphyrins for cultured cells and their subcellular distribution are dependent on their hydro-/lipo-solubility, e.g. as expressed by their octanol/water partition coefficient; thus, different cellular targets can be damaged by using porphyrins of different solubility as photosensitisers (Sandberg & Romslo, 1981).

In this paper, we extended the latter investigations to *in vivo* systems by studying the time-dependence of the subcellular localisation of a variety of porphyrins in liver and tumour cells of mice breaing a MS-2 sarcoma. The porphyrins injected were either dissolved in PBS and/or incorporated into small unilamellar liposomes of DPPC. We have previously shown (Jori *et al.*, 1983) that liposome transport of porphyrins such as Hp yields higher endocellular concentrations of the drug and enhances its preferential retention by tumour cells as compared with normal cells.

Correspondence: G. Jori.
Received 28 October 1985; and in revised form, 20 January 1986

### Materials and methods

### *Chemicals*

All porphyrins were obtained from Porphyrin Products (Logan, Utah, USA) and used as received; high pressure liquid chromatography (HPLC) analysis showed that the Hp sample contained ~5% protoporphyrin IX, 3–5% 2(4)-hydroxyethyl-4(2)-vinyl-deuteroporphyrin IX and traces of other unidentified porphyrin-type impurities. HpD (Photofrin I) was kindly supplied by Prof. T.J. Dougherty as a sterile aqueous solution, pH 7.4, at a nominal porphyrin concentration of $2.5\,mg\,ml^{-1}$. The concentration of the other porphyrin solutions was estimated spectrophotometrically using the following $\varepsilon$ values ($M^{-1}cm^{-1}$) (Marks, 1969): Hp, $4.23 \times 10^5$ ($0.25\,M\,H_2SO_4$, 401 nm); haematoporphyrin IX dimethylester (HpDME) $1.75 \times 10^5$ (pyridine, 402 nm); Uroporphyrin I (Up) $5.41 \times 10^5$ (0.1 M HCl, 405.5 nm); Uroporphyrin I octamethylester (UpOMe), $2.14 \times 10^5$ ($CHCl_3$, 406 nm); tetra(4-sulfonatophenyl)porphine (TPPS) $5.26 \times 10^5$ ($CH_3OH$, 415 nm). L-α-dipalmitoyl-phosphatidylcholine (DPPC) over 98% pure, was purchased from Sigma Chemical Co.; Sodium dodecylsulphate (SDS) and sucrose were analytical-grade reagents from Merck AG.

### *Animals and tumour*

Female mice of the BALB/c strain, 20 days old (30–40 g body wt), random bred in our laboratory, were used. The animals had free access to tap water and standard dietary chow. The tumour used was the MS-2 sarcoma, kindly supplied by Istituto

© The Macmillan Press Ltd., 1986

Nazionale dei Tumori, Milan. For routine passage, mice were injected s.c. in the dorsal region with $10^6$ viable cells suspended in 0.1 ml of PBS; viability was assayed by the trypan blue exclusion test. A solid tumour developed reaching a 0.71–1.0 cm diameter on the eight day after transplantation.

*Preparation of porphyrin-liposome systems*

Small unilamellar vesicles of DPPC, containing the porphyrin in a 1:70 molar ratio to the phospholipid were prepared immediately before use by the procedure previously described (Jori *et al.,* 1983). The homogeneity of the preparations was occasionally checked by chromatography of the liposomal suspensions in 0.01 M phosphate buffer at pH 7.4, containing 0.15 M NaCl, on a column (1.2 x 130 cm) of Sephacryl S-500; a mol. wt of 3.0–3.5 x $10^6$ was estimated for the liposomes. Electron microscopy analysis confirmed that the liposome preparations showed a reasonably homogeneous size distribution, the unilamellar vesicles having a diameter ranging between 33 and 35 nm. Prior to use, any unbound porphyrin was removed by gel filtration of the liposome suspension through a column of Sephadex G-100, where the liposomes are eluted at the void volume. The localisation of the porphyrin in either the phospholipid bilayer or the endoliposomal aqueous pool was established by fluorescence emission spectroscopy (Jori *et al.,* 1983).

*Pharmacokinetic studies*

The porphyrins, either dissolved in PBS or incorporated into unilamellar DPPC liposomes, were injected i.p. into tumour-bearing mice at 7 days after tumour transplantation. At this time, the tumour weight was about 70–80 mg, and increased to 95–110 mg at 10 days and to 120–140 mg at 13 days after transplantation. The injected dose was 5 mg prophyrin $kg^{-1}$ mouse body weight except in the case of liposomal Up and TPPS, where 1.5 mg $kg^{-1}$ were injected: the low efficiency of Up and TPPS incorporation into DPPC liposomes would have required the injection of too large volumes of liposomal suspension in order to reach a 5 mg $kg^{-1}$ concentration of porphyrin. At fixed time intervals, the mice were sacrificed, and four tissues (tumour, liver, kidneys and skin) were rapidly removed, washed with PBS to eliminate any surface blood, and homogenised in PBS. An aliquot of the homogenate was used for estimation of the porphyrin content by a spectrophotofluorimetric assay (Jori *et al.,* 1979) after extraction of the porphyrin from the tissue with 2% aqueous SDS. Control studies* indicated that at least 90% of the porphyrin present in the tissue is extracted by this procedure. The fluorescence data were converted into porphyrin concentration values by interpolation with a calibration plot built for each porphyrin in the presence of SDS micelles. A second aliquot of the tissue homogenate was used for cell fractionation by sucrose discontinuous gradient ultracentrifugation (Neville, 1976) after osmotic lysis of the cells. Three fractions were collected: the soluble fraction; the membrane fraction (37% sucrose); and the particulate fraction (41% sucrose, including the mitochondria, lysosomes and microsomes). The porphyrin content of each fraction was measured by the same procedure as used for tissues and normalised to one gram of nitrogen. The estimation of nitrogen content was performed according to Umbreit *et al.* (1959).

*Fluorescence measurements*

All fluorescence measurements were carried out by a Perkin Elmer MPF 4 spectrophotofluourimeter using quartz cuvettes of 1 cm optical path. Excitation was performed at 400 nm and the light emitted in the 550–750 nm region was recorded at a right angle to the incident beam. In preliminary experiments, we observed that the height of the two fluorescence emission peaks around 620 nm and

*The complete extraction of porphyrins from tissues is known to be a difficult process. In order to assess the reliability of our extraction procedure, we performed some preliminary experiments where three portions (~100 mg) of the liver of a normal mouse were assayed for the porphyrin content at 3 h after i.p. injection of Hp (5 mg $kg^{-1}$ body wt) by the following methods: portion 1, homogenisation with 10 ml of 2% aqueous SDS followed by 30 min incubation of the homogenate with the SDS micellar dispersion at 20°C under magnetic stirring; portion 2, homogenisation with 10 ml of 3 M HCl followed by 8 h hydrolysis of the homogenate with 3 M HCl at 60°C under nitrogen in sealed vials; portion 3, homogenisation with 7.5 ml of diethylether, centrifugation (10 min; 3,000 r.p.m.), collection of the ether layer, rehomogenisation of the tissue with 7.5 ml of acetic acid, centrifugation as above specified, collection of the acetic acid layer, extraction of the porphyrin from the combined ether/acetic acid layers with 3 M HCl. In all cases, the Hp content of the final solution was estimated by spectrophotofluorimetric analysis as specified in the experimental section. In 5 separate experiments (one mouse liver per experiment) the Hp recoveries obtained by the three different procedures never differed by more than 10%; moreover, re-extraction of residual Hp from the homogenised tissue by the same procedure yielded Hp recoveries which were about 5–10% the amount obtained by the first extraction.

680 nm, which are typical of porphyrins, is proportional to the integrated area below the whole emission spectrum; therefore, the sum of the emission intensities at the two aforesaid wavelengths was routinely used for the calculation of porphyrin concentration. Care was taken to keep the absorbance of the analysed solutions lower than 0.1 at 400 nm in order to minimise artifacts due to inner filter effects.

### Results

### *Porphyrin-liposome interactions*

Under our experimental conditions, all the porphyrins examined underwent incorporation into unilamellar DPPC liposomes. The spectroscopic parameters (see Table I) suggested that Hp, HpDME and UpOME were located as monomeric entities in the phospholipid bilayer, which is in agreement with the poor water-solubility of these porphyrins. Gel filtration studies on Sephadex G-100 columns demonstrated that the incorporation yield was essentially 100% in the case of the two porphyrin esters, and around 90% for Hp. On the other hand, Up and TPPS appeared to be located in the endoliposomal aqueous compartment; the low incorporation yield ($\sim$5%) of the two latter porphyrins is likely to reflect their statistical distribution between the internal and external aqueous pools.

The association of the porphyrin molecules with the DPPC liposomes was very stable, as shown by the slow rate of porphyrin leakage from the vesicles when DPPC liposomes containing 0.04 mM porphyrin were resuspended in PBS at 37°C: under these conditions, after 24 h about 10% Hp and no appreciable amount of HpDME were released. The addition of 0.2 mM human serum albumin had no effect on the release of Hp and HpDME from the DPPC liposomes. On the other hand, over 95% of Hp bound with 0.2 mM albumin in a 1:1 complex was captured by DPPC liposomes (the phospholipid concentration was 2.5 mg ml$^{-1}$).

In a few experiments, the interaction of HpD with DPPC liposomes was also studied. As shown in Table I, good incorporation efficiencies were obtained; however, the spectroscopic analyses pointed out that the liposome-bound porphyrin material was largely heterogeneous as regards both the localisation sites and the aggregation state.

### *Pharmacokinetics of aqueous and liposome-bound haematoporphyrin*

In Table II we report the recovery of Hp from different tissues of mice bearing a MS-2 sarcoma at various times after administration of the porphyrin either dissolved in PBS or incorporated into unilamellar DPPC liposomes. The data represent the average of Hp recoveries obtained by separate analyses of tissues from three different mice at each time; the largest deviation from the tabulated figures was $\pm$20%. The analyses were performed at particularly narrow time intervals during the initial 24 h, since this time period usually corresponds with the maximal porphyrin accumulation in liver and tumour tissues (Gomer & Dougherty, 1979; Jori *et al.*, 1979). On the other hand, it was not possible to extend the investigations beyond 6 days from Hp administration owning to the exceedingly large size of the tumour and possible death of the animals. In all cases, the extracted porphyrin samples exhibited identical spectroscopic properties independently of the location site. Previous studies (Tomio *et al.*, 1982) pointed out that Hp undergoes no appreciable metabolic alteration in normal and tumour-bearing animals. Column chromatographic analyses of serum samples taken from mice injected with aqueous or liposome-bound Hp were

**Table I** Fluorescence properties and endoliposomal localisation of various porphyrins associated with unilamellar DPPC vesicles.

| | *Fluorescence emission λ max (nm)*[a] | | | | |
|---|---|---|---|---|---|
| *Porphyrin* | *$H_2O$, pH 7.4* | *$CH_3OH$* | *Liposomal dispersion* | *Endoliposomal localisation* | *Incorporation yield (%)* |
| Hp | 614 | 625 | 625 | Lipid bilayer | >90 |
| HpDME | | 622 | 625 | Lipid bilayer | 100 |
| HpD | 612 | 618 | 610–620 | Heterogeneous | 90 |
| Up | 617 | 624[b] | 619 | Aqueous pool | 5 |
| UpOME | | 627 | 627 | Lipid bilayer | 100 |
| TPPS | 640 | 648[b] | 640 | Aqueous pool | 5 |

[a]The excitation wavelength was 400 nm. The porphyrin concentration was 10 μM in all cases; [b]In methanol containing 5% water.

**Table II** Recovery of hematoporphyrin (expressed as $\mu g$ porphyrin $g^{-1}$ of tissue) from selected mouse tissues at various times after i.p. injection of $5 mg kg^{-1}$ body wt aqueous or liposome-bound porphyrin to mice bearing a MS-2 fibrosarcoma.

| | *Liver* | | *Kidneys* | | *Skin* | | *Tumour* | |
|---|---|---|---|---|---|---|---|---|
| *Time* | *Hp-aq* | *Hp-lip* | *Hp-aq* | *Hp-lip* | *Hp-aq* | *Hp-lip* | *Hp-aq* | *Hp-lip* |
| control | 0.6 | 0.6 | 0.3 | 0.3 | 0.1 | 0.1 | 0.6 | 0.6 |
| 1 h | 4.7 | 3.6 | 2.1 | 1.2 | 0.8 | 0.4 | 1.3 | 0.9 |
| 2 h | 4.0 | 4.2 | 2.3 | 2.5 | 1.3 | 0.4 | 1.8 | 1.1 |
| 8 h | 3.1 | 5.3 | 2.0 | 2.5 | 1.7 | 2.0 | 2.1 | 1.8 |
| 12 h | 2.5 | 4.0 | 1.5 | 2.0 | 3.2 | 1.5 | 2.1 | 2.4 |
| 24 h | 1.5 | 3.2 | 1.0 | 1.4 | 1.7 | 1.3 | 1.9 | 4.1 |
| 72 h | 1.1 | 1.3 | 0.7 | 0.9 | 1.9 | 1.0 | 1.7 | 4.7 |
| 140 h | 0.7 | 0.9 | 0.5 | 0.8 | 0.8 | 0.5 | 1.0 | 2.9 |

performed at various times according to the procedure previously described (Jori *et al.*, 1984). In agreement with previous findings (Jori *et al.*, 1984), we observed that the aqueous porphyrin was initially distributed among at least three classes of serum proteins, *viz.* lipoproteins, globulins and albumins, but was completely eliminated from the two latter carriers within 48 h: at this time, only ~10% of the administered Hp was still bound with lipoproteins, especially high-density lipoproteins. On the other hand, less than 20% of the liposome-bound Hp became associated with globulins and albumins; a large fraction (70–80%) either remained in intact liposomes or was delivered to low- and high-density lipoproteins. The Hp-lipoprotein complex was very stable and its serum concentration decreased at a slow rate: about 35% of the originally administered Hp was still associated with the lipoprotein system after 48 h.

*Pharmacokinetics of various aqueous and liposome-bound porphyrins*

The recoveries of various porphyrins, administered to tumour-bearing mice either dissolved in PBS or bound with DPPC liposomes, were evaluated after 2 h and 48 h from injection. These time intervals were selected since they correspond with a large accumulation of porphyrins in most tissues (Figge *et al.*, 1948) and, respectively, the performance of photodynamic therapy of tumours in clinical applications (Dougherty, 1981). The averaged data from three mice analysed separately at each time (maximum deviation ± 15%) are shown in Table III. In general, under our experimental conditions, relatively high recoveries were obtained with all the porphyrin samples studied. Only in the case of liposome-bound Up and TPPS, low amounts of porphyrin were found to be present in all tissues at both 2 h and 48 h; this fact is probably a consequence of the small porphyrin concentrations which had to be injected (see experimental section). Once again, the spectroscopic properties of the porphyrins extracted from the tissues were identical with those of the uninjected porphyrins. Moreover, as one can see from Table II, the amount of endogenous porphyrins present in the tissues analysed by us was too low to interfere with the determination of the administered drugs.

**Table III** Recovery of various porphyrins (expressed as $\mu g$ porphyrins $g^{-1}$ of tissue) at 2 h and 48 h after i.p. injection of $5 mg kg^{-1}$ body wt aqueous or liposome-bound drug to mice bearing a MS-2 fibrosarcoma.

| | *Liver* | | *Tumour* | |
|---|---|---|---|---|
| *Porphyrin* | *2 h* | *48 h* | *2 h* | *48 h* |
| HpDME-lip | 4.5 | 2.1 | 2.3 | 5.7 |
| Up-aq | 3.9 | 1.0 | 4.2 | 0.8 |
| Up-lip[a] | 0.9 | 1.0 | 1.1 | 1.8 |
| UpOME-lip | 3.2 | 1.9 | 1.7 | 3.1 |
| TPPS-aq | 4.7 | 2.0 | 5.5 | 3.5 |
| TPPS-lip[a] | 0.8 | 1.2 | 0.5 | 1.7 |

[a]The injected dose was $1.5 mg kg^{-1}$ body wt.

In a few experiments, the pharmacokinetic studies above described were repeated with normal mice. In general, the recoveries of the aqueous or liposome-bound porphyrins from liver, kidneys and skin at 2 h and 48 h were fairly similar with those reported in Table III, indicating that the presence of the tumour exerts no major influence on the uptake and release of porphyrins from normal tissues.

*Studies on the subcellular distribution of porphyrins in mouse hepatocytes and tumor cells*

The subcellular distribution of the aqueous or

**Table IV** Subcellular distribution of various porphyrins in hepatocytes isolated from mice bearing a MS-2 fibrosarcoma at 2h and 48h after i.p. injection of $5\,mg\,kg^{-1}$ aqueous or liposome-bound drug. The recoveries are expressed as ng porphyrin $g^{-1}$ nitrogen.

| | *Subcellular fraction* | | | | | |
|---|---|---|---|---|---|---|
| | *Soluble* | | *Membrane* | | *Particulate* | |
| *Porphyrin* | *2h* | *48h* | *2h* | *48h* | *2h* | *48h* |
| Hq-aq | 0.35 | 0.17 | 0.19 | 0.40 | 0.07 | 0.10 |
| Hp-lip | 0.20 | 0.25 | 0.54 | 0.63 | 0.12 | 0.08 |
| HpDME-lip | 0.10 | 0.08 | 0.45 | 0.90 | 0.10 | 0.10 |
| Up-aq | traces | | traces | | traces | |
| Up-lip[a] | 0.21 | 0.17 | 0.08 | 0.05 | 0.17 | 0.10 |
| UpOME-lip | 0.05 | 0.05 | 0.80 | 0.37 | 0.10 | 0.12 |
| TPPS-aq | 0.17 | 0.23 | 0.05 | 0.11 | 0.14 | 0.11 |
| TPPS-lip[a] | 0.27 | 0.30 | 0.11 | 0.08 | 0.12 | 0.14 |

[a]The injected dose was $1.5\,mg\,kg^{-1}$ body wt.

**Table V** Subcellular distribution of various porphyrins in tumour cells isolated from mice bearing a MS-2 fibrosarcoma at 2h and 48h after i.p. injection of $5\,mg\,kg^{-1}$ body wt aqueous or liposome-bound drug. The recoveries are expressed as ng porphyrin $g^{-1}$ nitrogen.

| | *Subcellular fraction* | | | | | |
|---|---|---|---|---|---|---|
| | *Soluble* | | *Membrane* | | *Particulate* | |
| *Porphyrin* | *2h* | *48h* | *2h* | *48h* | *2h* | *48h* |
| Hp-aq | 0.48 | 0.21 | 0.38 | 0.57 | 0.12 | 0.12 |
| Hp-lip | 0.42 | 0.14 | 0.48 | 0.80 | 0.10 | 0.06 |
| HpDME-lip | 0.09 | 0.05 | 0.75 | 0.89 | 0.10 | 0.16 |
| Up-aq | traces | | traces | | traces | |
| Up-lip[a] | 0.30 | 0.36 | 0.14 | 0.10 | 0.05 | traces |
| UpOME-lip | 0.12 | traces | 0.58 | 0.72 | traces | |
| TPPS-aq | 0.91 | 0.79 | 0.11 | 0.07 | 0.12 | 0.05 |
| TPPS-lip[a] | 0.75 | 0.83 | 0.30 | 0.10 | 0.13 | 0.11 |

[a]The injected dose was $1.5\,mg\,kg^{-1}$ body wt.

liposome-bound porphyrins in the liver and tumour cells was estimated at 2h and 48h after drug administration. The data of porphyrin recovery, normalised to a standard nitrogen content for each fraction, are reported in Table IV (liver cells) and Table V (tumour cells), respectively.

### Discussion

The pharmacokinetic studies described in this paper confirm the preferential affinity of Hp for neoplastic as compared with normal tissues.* The tumour-localising properties of this prophyrin (see Table II) are remarkably enhanced when Hp is administered to tumour-bearing mice in association with unilamellar DPPC vesicles; this observation agrees with previous findings from our laboratory (Jori *et al.*, 1983). Actually, although transplanted tumours are known to contain a significant proportion of phagocytes which may uptake the liposomal vesicles, previous studies (Kessel & Chou, 1983; Cozzani *et al.*, 1984, 1985) showed that liposome-bound Hp and HpDME are accumulated by a variety of cultured malignant cells in remarkably larger amounts than by cultured normal cells. Therefore, liposomal transport appears to yield an improved loading of tumour cells with porphyrins. This fact is probably related to the inability of some serum proteins to extract Hp from the phospholipid bilayer of DPPC liposomes, where the porphyrin is located (Jori, 1985); it has been shown that serum albumin competes with cells for

*According to Dougherty (1981) the tumour-localising properties found for some commercial samples of Hp are associated with the presence of about 10–15% covalent dimers of the prophyrin.

Hp binding and accelerates the release of Hp from both normal and malignant cells (Cozzani et al., 1984). Moreover, DPPC liposomes, having a phase transition temperature of 41.5°C, exist in a quasi-solid state at the body temperature, hence they are internalised by cells mainly via endocytosis; as a consequence, the liposome-incorporated drug is released from inside the cell, probably when liposomes are opened at the lysosome level (Straubinger et al., 1983). Such a mechanism of Hp delivery from the liposomes to cells may justify both the relatively slow accumulation and the prolonged retention of liposomal porphyrins by tumour tissues. In particular, one should take into consideration the stable association of liposomal Hp with low- and high-density lipoproteins in the serum of mice; these proteins may act as a porphyrin source for the tumour up to at least 72 h after injection of the drug (Jori, 1985). Thus, significant amounts of Hp are still present in the tumour at 72 h and longer times after administration of the liposome-bound porphyrin, and tumour/liver ratios of Hp concentration as high as 3 are reached; on the other hand, this ratio is constantly below or around 1 when aqueous Hp is administered to mice bearing a MS-2 fibrosarcoma. The larger accumulation of Hp by tumour tissues should not be accompanied by an increase of the general cutaneous photosensitivity, which represents one major side effect of the photodynamic therapy of tumours (Dougherty et al., 1983): as one can see from Table II, the skin levels of Hp are fairly similar in the case of the aqueous and liposomal drug.

Table III shows that, also in the case of Up and TPPS, liposomal transport leads to a particularly stable association of the porphyrin with the tumour tissue. Actually, both liposomal Up and liposomal TPPS give quite similar porphyrin levels in the tumour between 2 h and 48 h after administration to mice, whereas the concentrations of the corresponding aqueous porphyrins in liver and tumour undergo a substantial decrease over the same time interval. The clearance is especially rapid in the case of aqueous Up, probably owing to the inability of this porphyrin to penetrate cells to any significant extent: as shown in Tables IV and V, only traces of aqueous Up are detected in all the subcellular fractions from liver and tumour examined by us. These observations may explain the poor photosensitising efficiency displayed by Up *in vivo* (Jori & Spikes, 1984). On the other hand, the lower rate of elimination of aqueous TPPS from tissues can be related with its significant localisation in the endocellular regions; this is also in agreement with the photocytotoxic effects observed after administration of TPPS to experimental animals (Grenan et al., 1980). Liposome-transported TPPS shows a subcellular distribution pattern closely similar with that found for aqueous TPPS; hence the partitioning of this porphyrin among the various cell compartments appears to be independent of the delivery mechanism. In general, the fate of porphyrins, once released inside the cell, appears to be controlled by their degree of hydro-/lipo-solubility. Actually, UpOME and HpDME, which are essentially water-insoluble, are almost exclusively recovered from the membrane fraction at both 2 h and 48 h after administration. A preferential localisation at the level of cell membranes is also observed for Hp, a relatively hydrophobic porphyrin. It is likely that this class of porphyrins are embedded into lipid cores of the membrane, thus becoming sparingly accessible to albumin and other serum proteins; the latter represent the usual carriers of porphyrins in the bloodstream. As known, porphyrin-photosensitisation of mammalian cells induces the formation of cholesterol hydroperoxides besides cross-linking of membrane proteins (Spikes, 1983), although the relative weight of either photoprocess in causing the membrane lysis is not definitely assessed as yet.

In any case, it is apparent that liposome carriers for porphyrins overcome the problems connected with both the blood transport of water-insoluble porphyrins and the hydrophobic membrane barrier preventing the cell penetration by highly polar porphyrins, such as Up. Therefore, the number of porphyrins potentially employable for the photodynamic therapy of tumours is enlarged; at the same time, the larger concentrations and longer retention of the drug thus obtained in the neoplastic area should determine an increased phototherapeutic efficiency and a greater flexibility in the definition of the porphyrin dose or in the choice of the optimal time interval between porphyrin administration and irradiation of the patient. Furthermore, the possibility exists to target different subcellular sites by selecting the porphyrin to be transported. This should not cause large modifications of the intrinsic efficiency of the photosensitisation process, since most free base porphyrins display similar photophysical properties, including the ability to generate the cytotoxic agent $^1O_2$ (Bonnett et al., 1980, 1983; Reddi et al., 1983). On the other hand, the preferential localisation of porphyrins in given subcellular structures can affect the type of photodamaged targets, hence the mechanism and kinetics of cell necrosis (Brun et al., 1981; Sandberg & Romslo, 1981). Again, this circumstance amplifies the potentialities of photodynamic therapy, especially as regards its possible extensions to the treatment of diseases other than tumours (Berns et al., 1984; Venezio et al., 1985).

This work received financial support from Consiglio Nazionale delle Ricerche (Italy) under the Special Project 'Oncologia', contract No. 84.00630.44.

### References

BERNS, M.W., RETTENMAIER, M., McCULLOUGH, J. & 5 others (1984). Response of psoriasis to red laser light following systemic injection of hematoporphyrin derivative. *Lasers Surg. Med.*, **4**, 73.

BONNETT, R., BERENBAUM, M.C. & KAUR, H. (1984). Chemical and biological studies on hematoporphyrin derivative: an unexpected photosensitization in brain. In *Porphyrins in Tumour Phototherapy*, Andreoni, A. & Cubbeddu, R. (eds) p. 87. Plenum Press: New York.

BONNETT, R., CHARALAMBIDES, A.A., LAND, E.J., SINCLAIR, R.S., TAIT, D. & TRUSCOTT, T.G. (1980). Triplet states of porphyrin esters. *J.C.S. Faraday I*, **76**, 852.

BONNETT, R., LAMBERT, C., LAND, E.J., SCOURIDES, P.A., SINCLAIR, R.S. & TRUSCOTT, G. (1983). The triplet and radical species of hematoporphyrin and some of its derivatives. *Photochem. Photobiol.*, **38**, 1.

BRUN, A., HODVING, G. & ROMSLO, I. (1981). Protoporphyrin-induced photohemolysis: differences related to the subcellular distribution of protoporphyrin in erythropoietic protoporphyria and when added to normal red cells. *Int. J. Biochem.*, **13**, 225.

COZZANI, I., JORI, G., BERTOLONI, G. & MILANESI, C. (1985). Efficient photosensitization of malignant human cells in vitro by liposome-bound porphyrins. *Chem. Biol. Interactions*, **53**, 131.

COZZANI, I., JORI, G., REDDI, E., TOMIO, L., SICURO, T. & MALVADI, G. (1984). Interaction of free and liposome-bound porphyrins with normal and malignant cells. In *Porphyrins in Tumour Phototherapy*, Andreoni, A. & Cubbeddu, R. (eds) p. 157. Plenum Press: New York.

DOUGHERTY, T.J. (1980). Hematoporphyrin derivative for detection and treatment of cancer. *J. Surg. Oncol.*, **15**, 209.

DOUGHERTY, T.J. (1981). Hematoporphyrin as a photosensitizer of tumors. *Photochem. Photobiol.*, **38**, 377.

DOUGHERTY, T.J. (1981). Photoradiation therapy for cutaneous and subcutaneous malignancies. *J. Invest. Dermatol.*, **77**, 122.

DOUGHERTY, T.J., BOYLE, D.G., WEISHAUPT, K.R., HENDERSON, B.A., POTTER, W.R. & WITYK, K.E. (1983). Photoradiation therapy. Clinical and drug advances. In *Porphyrin Photosensitization*, Kessel, D. & Dougherty, T.J. (eds) p. 3. Plenum Press: New York.

FIGGE, F.H.J., WEILAND, G.S. & MANGANIELLO, L.O.J. (1948). Cancer detection and therapy. Affinity of neoplastic, embryonic and traumatized tissues for porphyrins and metalloporphyrins. *Proc. Soc. Expl. Biol. Med.*, **68**, 640.

GOMER, C.J. & DOUGHERTY, T.J. (1979). Determination of $^{3}H$-$^{14}C$-hematoporphyrin derivative distribution in malignant and normal tissue. *Cancer Res.*, **39**, 146.

GRENAN, M., TSUTSUI, M. & WYSOR, M. (1980). Phototoxicity of the chemotherapeutic agents hematoporphyrin D, meso-tetra(p-sulfophenyl)-porphine, and zinc tetra(p-sulfophenyl)porphine. *Res. Commun. Chem. Pathol. Pharmacol.*, **30**, 317.

JORI, G. (1985). Pharmacokinetic studies with hematoporphyrin in tumor-bearing mice. In *Photodynamic Therapy of Tumors and Other Diseases*, Jori, G. & Perria, C.A. (eds) p. 159. Lib. Progetto: Padova.

JORI, G., BELTRAMINI, M., REDDI, E., SALVATO, B., PAGNAN, A. & TSANOV, T. (1984). Evidence for a major role of plasma lipoproteins as hematoporphyrin carriers in vivo. *Cancer Lett.*, **24**, 291.

JORI, G., REDDI, E., TOMIO, L., SALVATO, B. & ZORAT, P.L. (1979). Time dependence of hematoporphyrin distribution in selected tissues of normal rats and in ascites hepatoma. *Tumori*, **65**, 425.

JORI, G. & SPIKES, J.D. (1984). The photobiochemistry of porphyrins. In *Topics in Photomedicine*, Smith, K.C. (ed) p. 183. Plenum Press: New York.

JORI, G., REDDI, E., ROSSI, E. & CORTI, L. (1983). Preferential delivery of liposome-incorporated porphyrins to neoplastic cells in tumor-bearing rats. *Br. J. Cancer*, **48**, 307.

KESSEL, D. (1981). Transport and binding of hematoporphyrin derivative and related porphyrins by murine leukemia L1210 cells. *Cancer Res.*, **41**, 1318.

KESSEL, D. & CHOU, T.H. (1983). Porphyrin localizing phenomena. In *Porphyrin Photosensitization* Kessel, D. & Dougherty, T.J. (eds) p. 115. Plenum Press: New York.

MOAN, J., JOHANNESSEN, J.V., CHRISTENSEN, T., ESPEVIK, T. & McGHIE, J.B. (1982). Porphyrin-sensitized photoinactivation of human cells in vitro. *Am. J. Pathol.*, **109**, 184.

NEVILLE, D. (1976). The preparation of cell surface membrane enriched fractions. In *Biochemical Analysis of Membranes*, Maddy, A.H. (ed) p. 27. Chapman and Hall: London.

REDDI, E., JORI, G., RODGERS, M.A.J. & SPIKES, J.D. (1983). Flash photolysis studies of hemato- and copro-porphyrins in homogeneous and microheterogeneous aqueous dispersions. *Photochem. Photobiol.*, **38**, 639.

SANDBERG, S. & ROMSLO, I. (1981). Porphyrin-induced photodamage at the cellular and subcellular level as related to the solubility of the porphyrin. *Clin. Chim. Acta*, **109**, 193.

SPIKES, J.D. (1983). Potentials of photosensitization in mammalian cells. In *Photoimmunology*, Parrish, J.A. & 2 others, (eds) p. 23. Plenum Press: New York.

STRAUBINGER, R.M., HONG, K., FRIEND, D.S. & PAPAHADJOPOULOS, D. (1983). Endocytosis of liposomes and intracellular fate of encapsulated molecules. *Cell*, **32**, 1069.

TOMIO, L., ZORAT, P.L., JORI, G., SALVATO, B. & REDDI, E. (1982). Elimination pathway of hematoporphyrin from normal and tumor-bearing rats. *Tumori*, **68**, 283.

UMBREIT, W.W., BURRIS, R.H. & STAUFFER, J.F. (1957). *Manometric techniques*, p. 274. Burgess Publishing Co.: Minneapolis.

VENEZIO, F.R., DIVINCENZO, C., SHERMAN, D. & 4 others (1985). Bactericidal effects of photoradiation therapy with hematoporphyrin derivative. *J. Infect. Dis.*, **151**, 166.